# Evolution of a programme to engage school students with health research and science in Kenya

**DOI:** 10.12688/wellcomeopenres.15106.1

**Published:** 2019-02-28

**Authors:** Alun Davies, Nancy Mwangome, Betty Yeri, Grace Mwango, Noni Mumba, Vicki Marsh, Dorcas Kamuya, Sassy Molyneux, Samson Kinyanjui, Caroline Jones

**Affiliations:** 1 Health Sysytems and Research Ethics, Center for Geographical Medicine, KEMRI-Wellcome Trust Research Programme, Kilifi, 80108, Kenya; 2 Centre for Tropical Medicine and Global Health, Nuffield Department of Medicine, University of Oxford, Oxford, UK; 3 IDEAL, Center for Geographical Medicine, KEMRI-Wellcome Trust Research Programme, Kilifi, Kenya; 4 Department of Biochemistry, Pwani University, Kilifi, Kenya

**Keywords:** Community, Public Engagement, health research, schools, science, PAR, scale-up

## Abstract

Facilitating mutually-beneficial educational activities between researchers and school students is an increasingly popular way for research institutes to engage with communities who host health research, but these activities have rarely been formally examined as a community or public engagement approach in health research. The KEMRI-Wellcome Trust Research Programme (KWTRP) in Kilifi, Kenya, through a Participatory Action Research (PAR) approach involving students, teachers, researchers and education stakeholders, has incorporated ‘school engagement’ as a key component into their community engagement (CE) strategy.

School engagement activities at KWTRP aim at strengthening the ethical practice of the institution in two ways: through promoting an interest in science and research among school students as a form of benefit-sharing; and through creating forums for dialogue aimed at promoting mutual understanding between researchers and school students.

In this article, we provide a background of CE in Kilifi and describe the diverse ways in which health researchers have engaged with communities and schools in different parts of the world. We then describe the way in which the KWTRP school engagement programme (SEP) was developed and scaled-up. We conclude with a discussion about the challenges, benefits and lessons learnt from the SEP implementation and scale-up in Kilifi, which can inform the establishment of SEPs in other settings.

## Background

Community and public engagement with health research is becoming widely acknowledged and described in the literature, and engagement with schools (school engagement) is becoming increasingly adopted by health research institutions as a component of their engagement programmes. Despite this, descriptions of school engagement approaches and their evaluations are rarely described in the academic literature, particularly in low and middle income countries (LMICs), and descriptions of how programmes were initiated and scaled-up are even rarer. We aim to address this gap through providing a description of the 10-year evolution of a School Engagement Programme (SEP) within the context of a large health research institution in Kenya.

### The general aims and approaches to community engagement in LMICs

In recent years an increasing emphasis has been placed on the importance of engagement between health researchers and communities who host research ^1– 5^. Community engagement (CE) with health research comes in a range of shapes and forms, and has a range of, sometimes conflicting goals, often addressing ethical principles of research ^6^. These goals comprise ^7^:

- Broadly protecting communities in research
- Minimising possible exploitation
- Increase the likelihood that research will generate fair benefits locally
- Ensure awareness and respect for local cultural differences
- Ensure respect for recruited participants and study populations
- Legitimacy of engagement process
- Partners share the responsibility of research
- Minimise community disruption
- Ensure that disparities, inequalities and stigma are not inadvertently replicated or reinforced

It is perhaps unsurprising that this range of goals have spawned several approaches to engaging host communities with health research. In Africa, in an attempt to address these goals, researchers and CE practitioners have engaged communities in a range of ways, including: ‘town-hall’ meetings with communities or stakeholders; focus group discussions; community advisory boards and deliberative sessions ^8^.

### The KEMRI-WT programme in Kenya

The KEMRI-Wellcome Trust Research Programme ( KWTRP) is a well-funded health research programme, that was established in the rural coastal town of Kilifi, in Kenya in 1989, as a collaboration between the Kenyan Medical Research Institute (KEMRI), the Wellcome Trust and the University of Oxford. Historically the focus of the KWTRP was on malaria research, but since 1989 the Programme has expanded substantially both in terms of research focus, diversifying to include basic biological research with clinical trials, epidemiology, social and behavioural sciences and health systems and policy research, and in geographical scope, establishing hubs in Nairobi, Kenya, and Mbale in Uganda. In early 2017 KWTRP employed 800 staff, in the Kilifi and Nairobi hubs, mostly Kenyan, but with a few research staff from other African countries and other parts of the world. The main hub of the KWTRP located in Kilifi town, the administrative capital of Kilifi County situated some 60km north of Mombasa, comprises training and administration facilities and state of the art biomedical laboratories.

The centre aims to: “Conduct research to the highest international scientific and ethical standards on the major causes of morbidity and mortality in the region, in order to provide the evidence base to improve health”; and “Train an internationally competitive cadre of Kenyan and African research leaders to ensure the long term development of health research in Africa”. By December 2018, the KWTRP had produced 103 completed PhDs, with many of these researchers currently employed as post-doctoral students and principal investigators in the programme and beyond.

Juxtaposed with this research wealth and hub of opportunities for further education are the educational and resource challenges faced by local communities in Kilifi County. Kilifi County, one of Kenya’s 47 administrative counties, is among Kenya’s *‘20 most marginalised counties’* ^9^, with 64% of its residents living in dwellings with earth floors. The County’s residents are mainly dependent on agriculture, tourism and fishing for employment and food ^10^, and 36% of Kilifi residents have no formal education, with only 13% having secondary school education and above, compared to 25% and 23% respectively across Kenya ^11^. Much of the epidemiological and clinical research conducted within the programme is underpinned by the Kilifi Heath and Demographic Surveillance System ( KHDSS), which collects demographic information about a population of 280,000 within a geographically defined area of 900km2, through regular visits to residential homesteads.

### CE at the KWTRP

In the early 2000s, a consultative process was embarked upon, drawing on inputs from local and international community and research stakeholders, to establish a communication strategy for the programme ^12^ aimed at strengthening communication and building mutual understanding between researchers and residents of the Kilifi KDHSS. For practical purposes, the KWTRP’s communication strategy defined ‘the community’ as the residents living within the KHDSS where the majority of the KWTRP’s research activities have been conducted ^12^.

CE in Kilifi has been divided into two broad mutually supportive components: study specific; and programme-wide engagement ^6^. Study specific engagement is aimed at addressing the range of communicational and engagement requirements of specific research studies, such as providing specific trial/study information to communities to support informed consent ^13– 15^ and disseminating specific research findings ^16^. Programme-wide engagement aims at addressing a broader range of ethical goals which cut across a range of studies, institutional practice and policy, and the basic principles of research. For example, programme-wide engagement activities have included strengthening the community’s understanding of research and gaining community feedback about institutional policies ^6^. Until 2010, engagement approaches have comprised community and stakeholder meetings; focus group discussions; deliberative sessions; open-days; and regular meetings with a network of 170 KEMRI Community Representatives elected by the community ^12, 17– 21^.

In 2005 there were 4 full-time staff employed to implement the CE strategy and by 2018 the team had grown to 20 staff making up what is now referred to as the community liaison group (CLG). The CLG coordinates and implements all programme-wide engagement at KWTRP and supports study-specific activities. The group draws on support from four senior social scientists.

From the outset, during open days and meetings, community members frequently requested a school engagement element to the work, specifically to provide careers advice and motivation to secondary school students wishing to pursue medical and scientific careers ^22^. In 2010, we initiated our school engagement work piloting our approach with three local secondary schools. In the following sections, we first provide an overview of the international experience and approaches to engaging school students with health research. We then go on to share our own experience, which evolved in parallel to many of the approaches described. Finally, we conclude with some key elements across the Kenyan experience which offer key considerations for future similar projects.

## International experience of school engagement

### Engagement between researchers and schools

Motivated by the growing evidence of the influence of ‘out-of-school-science’ in promoting positive attitudes towards science among school students ^23– 26^, and the need to engage with a range of community members, health researchers worldwide have sought several ways of engaging with local schools. A common theme for many school engagement with science approaches is that they anticipate that students will adopt scientists as role-models to look up to and emulate ^27– 33^. The adoption of science role-models in turn, has the potential to: inspire student career choices; challenge stereotypical perceptions of scientists; provide realistic insights into real-world science ^30^; and support science teachers to maintain students’ interest in the pursuit of science ^27^. Several initiatives ^28, 29, 31, 32^ have drawn inspiration from the ‘possible selves’ theory ^34^. In this theory, as children grow, their career aspirations develop as a result of their exposure to different careers, and influential individuals within careers. The breadth of children’s repertoire of possible future careers (or possible selves) can be widened when exposure to specific careers enables a belief that they are capable of achieving this career. Angela Porta ^31^, for example, reported that encounters with female biomedical researchers from diverse ethnic backgrounds challenged students’ stereotypical preconceptions of scientists, whilst other studies reported that interactions with scientists influenced their desire to become a scientist and promoted positive attitudes towards science ^28, 29, 32^.

Engagement between health researchers and schools has focussed mainly on educational goals, including: promoting an interest in health, science and science related careers; promoting science role models; promoting positive attitudes towards science; and de-mystifying science and scientists ^35– 38^. Less emphasis has been placed on CE goals such as: promoting an awareness of research; and feeding unique student perspectives into research implementation ^39– 43^; or a combination of both educational and CE goals ^22, 39^.

Approaches for engagement between health researchers and schools can be classified into four main types ^39^: a) School-Scientist partnerships, which have been popular in Australia, New Zealand and the USA since the 1980s, and involve scientists spending periods of time at schools (often several years) conducting activities aimed at promoting an interest in, and positive attitudes towards science, and in some cases collecting scientific data ^37, 44– 47^; b) Science work-experience attachments, where students spend extended periods attached to researchers at research institutions, learning about research careers and ‘science citizenry’ ^35, 48– 50^; c) Young Persons Advisory Groups, which consist of regular meetings between health researchers and groups of 10–15 school children, aimed at discussing and advising on research questions, procedures, implementation, disseminating of findings and the appropriateness of language and content of informed consent forms ^41– 43^; and d) Short encounters between researchers and schools, usually conducted through one-day (or less) events.

Several universities and science research institutes have reported on their experience with facilitating short, often one-day, interactions between researchers and school students. A prominent set of activities involves inviting school students to institutions to meet scientists and see laboratories ^22, 40, 51, 52^, or to attend open days comprising science demonstrations facilitated by scientists ^53^. An alternative approach in the USA is the ‘Scientists In Classrooms’ (Fitzakerley, Michlin ^54^ where neuroscientists enter classrooms to provide a 40–60-minute talk about their work aiming to promote neuroscience literacy and positive attitudes towards neuroscience. All these approaches are broadly aimed at raising an interest in science, demystifying the work of scientists, and raising awareness of research.

In Africa, there are a growing number of science centres targeting audiences of school students, particularly in Southern Africa ^55^. Of note is the SAASTEC programme (The Southern African Association of Science and Technology Centres), linked to South African Universities, which has initiated a network of Science Centres aimed at enhancing school students experience of learning science. The focus of these centres however, is a much broader engagement with science, as opposed to health research. Narrowing down to a focus on health research, several African research institution websites describe different engagement activities with local schools. Examples include: the MRC in The Gambia describe hosting school students to their centre; H3 Africa have an on-line platform where students can ask questions to scientists; and a South African project engages primary school students with health and research through popular music. However, despite this growth in engagement activities, published articles describing the purpose of such engagement, and evaluations of the outcomes of engagement between health research and schools in Africa, is restricted to less than a handful ^22, 39, 56^.

### Researcher gains from school engagement

Several studies describe factors that make school engagement challenging to researchers, for example having to work within the constraints of the school timetable ^37, 44, 57^, generally negative perceptions of engagement ^58^ and a common perception among scientists that engagement is done by those who are not good enough for science careers ^58– 60^. Contrary to the latter belief, a study involving data from 11,000 scientists, found a statistically significant correlation between public engagement activity and academic output ^59^. The authors of this study argue that dissemination activities (including popularisation of science in schools) do not compete with academic achievement, but the two are mutually supportive, contributing to a broadening of scientists’ horizons and generating new perspectives and ideas for research.

Descriptions of engagement between researchers and schools, report that participating scientists gained satisfaction and enjoyment from promoting science ^37, 47, 52, 61^ and that it contributed positively to their communication skills ^22, 37, 47, 52, 61^. Researchers have reported that engagement can offer insights into the context in which they work ^22, 37, 44, 62^, an appreciation of the challenges of working with schools including the heavy workloads of teachers ^37, 44^, and a better understanding of community knowledge of and attitudes towards their research ^22, 47, 61^. In Kenya, a low-income country, researchers reported that participating in school engagement offered them an opportunity to ‘give back’ to the community and contribute to local development through promoting science education ^22^. Engagement leading to gains in researchers’ appreciation of local concerns about research in LMICs has been highlighted as important, to inform better and more ethically sound research designs and implementation ^63^. With respect to engaging school students, the recent emergence of Young Persons’ Advisory Groups underscores the view that young people and adolescents have unique insights which can feed into research planning and implementation.

## Methodological approach to establishing a School Engagement Programme at KWRTP

Establishing a School Engagement Programme (SEP) in Kilifi was motivated by two factors. Firstly, by frequent requests by community leaders, for support in local schools: *‘What is KWTRP doing to advise our schoolchildren on what subjects to choose to become scientists?’* (Roka village chief, annual debriefing workshop, 25 October 2007 cited in Davies, Mbete ^22^). The second motivation came from KWTRP researchers’ desire to draw on its existing human and laboratory resources to enhance local students’ educational experiences and provide opportunities to and learn about science ^22, 64^. The latter acknowledged the relative wealth and resource disparity between the state-of-the-art research institution and local public secondary schools, often characterised as having large class-sizes, poorly resourced laboratories ^65, 66^ and infrequent opportunities for students to conduct practical science ^65^. School science education, not only in Kenya, but generally, often presents an abstract and artificial depiction of ‘real-world’ science where everything takes place in the confines of the school laboratory ^23, 24^. In poorly-resourced LMIC school laboratories with limited opportunities for experiments and observations, this abstractness is likely to be heightened. Based on the potential for “out-of-school” science experiences, (e.g. field visits) to contribute to more “authentic” school science ^23^, we felt that exposure to Kenyan scientists and locally conducted research, could benefit students through promoting an interest in, and positive attitudes towards school science, which could ultimately lead to improved school science achievement ^67– 70^. Benefitting school students in this way, combined with raising an understanding of locally conducted research aimed at addressing the ethical principles of research outlined in the Belmont Report ^71^; beneficence, justice, and respect for persons.

Whilst raising students’ interest in science and research related careers, it was felt that engaging schools presented a further opportunity to extend engagement with the broader community. Engaging with school students, though potentially important in its own right as described above, was also based on a premise that if young people can influence peer and family health-related beliefs and behaviour ^72– 76^, when exposed to researchers, they may be provided with opportunities to re-evaluate prevailing community knowledge, misconceptions, beliefs and attitudes related to health research, and influence community attitudes based on a fuller understanding of research.

### A participatory action approach to establishing a SEP

In 2008, the Wellcome Trust’s International Engagement Award provided funding to pilot a SEP as part of the KWTRP CE activities ^64^. A participatory action research (PAR) process was used from the outset to initiate and develop the SEP incorporating the views, ideas and needs of students, teachers, parents, county education officers and researchers. A PAR approach was chosen because of its strength in engaging the voices, perspectives and experiences of all the participants and researchers involved ^77, 78^. According to Baum, MacDougall ^79^ PAR *“focuses on research whose purpose is to enable action. Action is achieved through a reflective cycle, whereby participants collect and analyse data, then determine what action should follow. The resultant action is then further researched and an iterative reflective cycle perpetuates data collection, reflection, and action as in a corkscrew action.”* The development of the Kilifi SEP involved three cycles of PAR over a ten-year period, each entailing: brain storming and planning meetings with researchers, teachers, students and county education office staff; implementation and evaluation; and feedback/reflection sessions. The learning gained from each cycle fed into the planning and implementation of subsequent PAR cycles. Guiding this process was a shared understanding among the participants that the school engagement programme should be aimed at addressing both educational as well as engagement goals, specifically: promoting mutual understanding between researchers and the community; nurturing respect for the community among researchers; promoting an interest in and positive attitudes towards science and science related careers among students (as a means of benefit sharing); and raising awareness of locally conducted research ^22^.

### PAR cycle 1: 2009 – 2010

Developing the initial pilot SEP activities in 2009 involved convening separate group discussions with teachers and students from three schools, and researchers, in order to assess willingness and gather ideas for engagement. Information and ideas from these discussions were then compiled and fed into an initial 2-day workshop involving teachers and researchers. At the time, based on a consensus between teachers, researchers and education office staff, students were not included in this workshop, because it was felt that their free communication would be hindered by the presence of teachers and participating researchers (this decision was revised to include students in the second and third PAR cycles). The 2009 workshop comprised three components: a) learning about research through a KWTRP tour and interactive activities with researchers; b) brainstorming potential engagement activities; and c) ranking the brainstormed ideas, based on their perceived value for students and implementability. The school engagement activities developed through this process were implemented in the three participating schools between May and August 2009 and comprised: school visits to KWTRP for a lab tour and interactive sessions with researchers; researcher visits to schools to give careers talks; and science-based competitions for students. An evaluation of this pilot programme, using mixed methods including: pre and post intervention student surveys; focus group discussions; and in-depth interviews with students, teachers and education officers, found that the activities promoted a better understanding of, and positive attitudes towards, health research and school biology among students ^22^. Further, the activities were well-received by parents, teachers and education officers, and that engagement provided researchers with an appreciation of the context in which they worked ^22^. During feedback meetings with teachers, the findings of the evaluation were combined with teacher and researcher experiences to inform the development of further ideas for engagement. These ideas were implemented in the second PAR cycle of the SEP’s development.

### PAR cycle 2: 2010 – 2012

Following the success of the initial two years of the SEP, the Wellcome Trust provided funding for a continuation of activities from 2011 to 2012. The second PAR cycle incorporated feedback and reflection from the first PAR cycle into a new series of brainstorming and planning discussions with teachers, researchers and students. This enabled: the scale-up of the SEP to five secondary schools, the inclusion of activities to support school science clubs in preparation for the national School Science and Engineering Fair (SEF) competition; and the establishment of a 3-month attachment scheme at KWTRP for nine top-performing school leavers from Kilifi County per year.

In 2012, SEP activities involved an estimated 1000 students visiting the KWTRP for engagement activities, with more students interacting with researchers through researcher visits to schools to give career and inspirational talks.

### PAR cycle 3: 2013 – 2017

**Figure 1.**
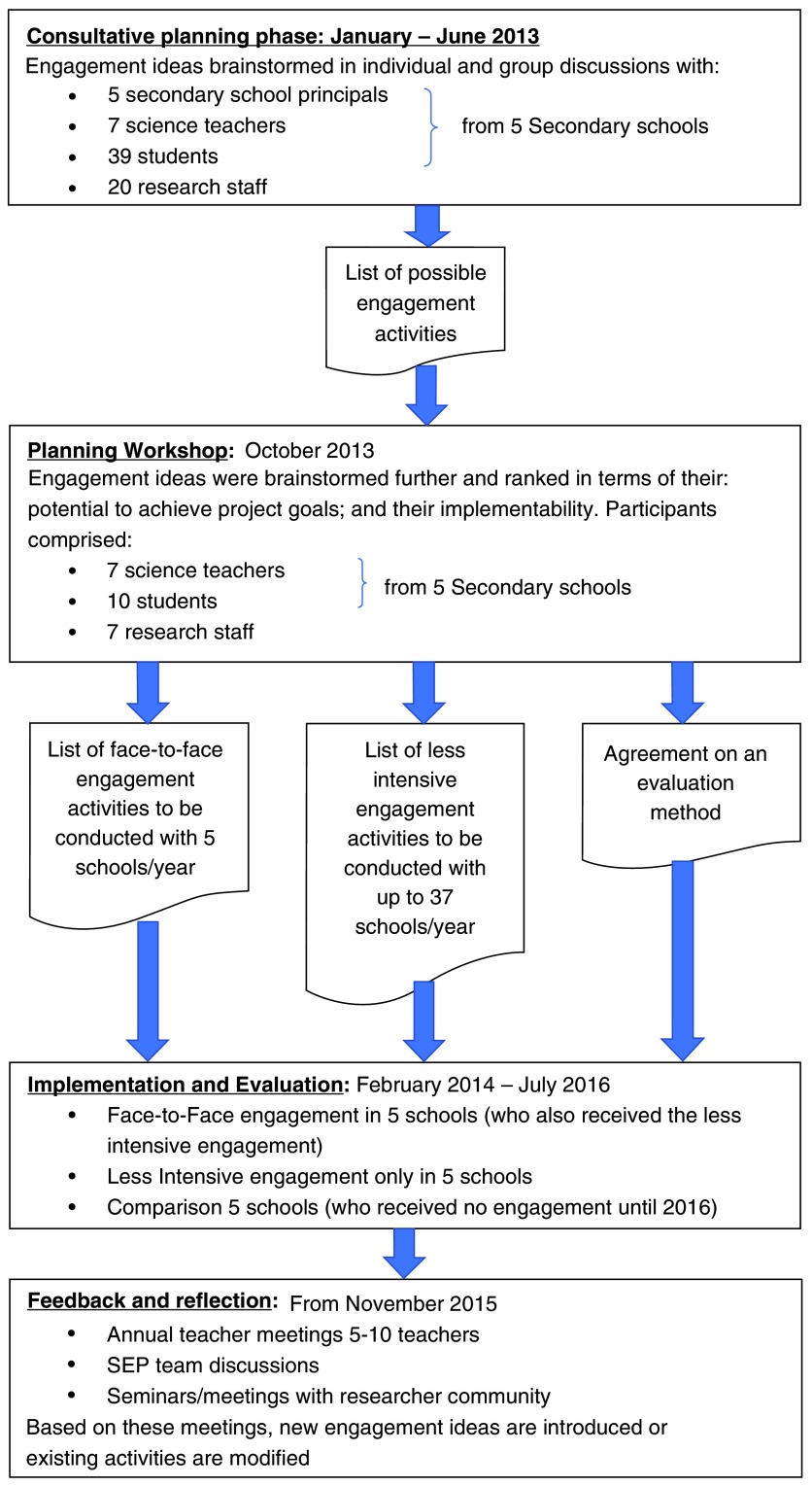
Phase 3 of the PAR cycle.

Based on the findings of the pilot evaluation (Davies, Mbete *et al.* 2012), participant feedback and reflections on PAR cycles 1 and 2, a demand for inclusion from other schools in Kilifi County, and a desire among the KWTRP’s CLG to scale-up engagement with all 37 state secondary schools across the KDHSS area, a third round of funding was acquired from the Wellcome Trust’s International Engagement Award ^80^. Scaling up the SEP to 37 schools raised two main constraints: a) the limited number and time of researchers to engage with schools; and b) the limited capacity of the KWTRP laboratories to host visiting school groups without excessively disrupting the day-to-day research activities. In 2013 a third cycle of PAR was conducted to help ensure that the expansion was planned in a way that supported effectiveness, efficiency and sustainability from both school and research stakeholders’ perspectives. This process is described in Figure 1 below and resulted in two broad approaches for engagement: a) concentrated face-to-face engagement with 5–6 schools per year on a rotational basis using engagement activities developed between 2008 – 2012; and b) a less-intensive engagement with activities which could be conducted with 37 schools. These activities are summarised in Table 1 below. A component of the less intensive engagement was web-based. Students with internet access could engage with research through an interactive SEP website, which documents SEP activities, provided science resources and introduces students to a range of research staff, and through an on-line engagement programme called “I’m a scientist get me out of here” (IAS). Over the two-week IAS implementation period in 2014, an estimated 200 students from 10 schools asked 5 participating scientists nearly 500 questions related to science and health. The site received over 10,000 hits over the event. Between 2014 and 2018, these face-to-face and less-intensive engagement activities were rolled out over a total of 37 schools and evaluated using a mixed method approach, and this is described elsewhere ^39^.

**Table 1.** Summary of school engagement programme activities conducted between 2015–2017.

Concentrated face-to-face engagement 5–6 different schools a year	Less Intensive engagement 37 schools a year
1. Form 1 & 2 student KWTRP lab tour and interactive sessions with research staff 2. Science club visits to KWTRP – students present SEF projects to researchers’ and receive feedback 3. Scientist visits to schools to discuss research and their careers. 4. Inclusion in Engagement B activities	1. On-line engagement through: • The IAS platform • The KWTRP-SEP website 2. Annual Science Symposium (quiz) for teams of 4 students from 37 schools

As well as developing and agreeing on new approaches and activities for engagement, workshop participants were able to contribute ideas towards evaluating the activities. The evaluation, described elsewhere (manuscript in preparation), enabled further reflection among the SEP team, teachers and researchers, and this continuing cycle enables the SEP to be responsive to the needs of the participants.

SEP activities are voluntary for schools with all costs covered by the KWTRP. The decision by the school principal to allow their school to participate in individual SEP activities is influenced by several factors. These factors include: school participation in other extracurricular activities; time pressure for teachers to complete specific subject syllabi; and specific to IAS participation, the availability of computers and internet connectivity in the school. Though resources in Kenyan Secondary schools are limited ^65^, in 2006 the government of Kenya launched a schools Information Technology policy ^81^ and access to computers has grown steadily in schools through the support of several international partners ^82^. In 2014 IAS, was not accessible to all schools, however this is likely to improve in the future if the Kenya government adheres to its commitment to improve ICT infrastructure in schools and equip students with IT skills ^83^.

## Discussion

This paper has provided an overview of the ways in which researchers have engaged with schools, and a description of how a PAR approach involving researchers, teachers, students and county education officers was used to establish the SEP at KWTRP in Kilifi, Kenya. The PAR approach generated a programme of activities facilitating engagement and interaction between KWTRP researchers and students and guided the scale-up of the programme from 3 to 37 local secondary schools. Combining two approaches for engaging schools enabled a ‘concentrated engagement’ on a rotational basis with 5 school per year, whilst maintaining contact with 32 schools through ‘less-intensive engagement.’ Over a 6-year period between 2012 and 2018 an estimated 1000 students per year from 37 schools have participated in ‘concentrated face-to-face engagement’ and an additional 2000 students participated in ‘less-intensive engagement’ per year in 2017 and 2018. Further, on-line engagement has enabled the extension of engagement to schools beyond Kilifi, in Nairobi, Nakuru and Kisumu.

Clearly establishing a SEP cannot rely solely on the goodwill of researchers, teachers and research institutions, it requires funding to sustain the activities, particularly in a context where schools have very little resources to support out-of-school activities. From 2009–2017, the Kilifi SEP has depended on the support of two extended Wellcome Trust International Engagement Awards worth £316,000 which has supported team salaries (one coordinator and two Community Liaison officers) and all school activities. Based on the success of the programme and continued support from the Kilifi County Education Office and the Wellcome Trust, the SEP secured funding for 5 years to expand the programme between 2017–2021.

The co-planned and co-implemented activities aimed at combining educational goals with goals of community engagement. At the outset it was envisioned that interactions between students and researchers would: nurture students’ interest in science, awareness of science related careers, and understanding of locally conducted health research; whilst researchers would gain valuable insights into community views, which would in turn, nurture a respect for the community hosting KWTRP’s research. Clearly, exploring whether these goals have been met requires rigorous evaluation (described elsewhere), however, demand for inclusion and continued participation from 37 schools and research staff, and continued support from the Kilifi County Education office, would suggest that at the very least, schools and researchers perceive the SEP activities to be beneficial. Reflecting on the demand for, and continued support for the SEP, implementing staff felt that including teachers, students and researchers in a PAR process, nurtured participant ‘buy-in’ and contributed to the activities’ appropriateness and uptake.

Tindana, Singh ^5^ describe how forming ‘authentic partnerships’ through CE can generate mutual benefits, or ‘win-win’ outcomes for researchers and communities. The importance of engagement generating mutual benefits has been re-enforced in more recent literature, see for example the report of the Participants in the CE and Consent Workshop ^6^. Arguably, what sets school engagement apart from other forms of CE is its unique potential for generating ‘win-win’ outcomes for participating researchers and students: as students get opportunities to learn about science and related careers, researchers gain from gaining valuable insights from school students, which can potentially be incorporated into research implementation ^84^ and an understanding of the context in which they work ^22^. The type of inferred benefits accrued through engagement, as experienced through the SEP, can create demand for further engagement among schools and researchers, thus enabling further opportunities to address CE goals. In this way, school engagement becomes ‘demand-driven’ as opposed to some other forms of potentially ‘supply driven’ engagement, with a greater focus on, for example, providing information about research for recruitment.

Whilst demand for inclusion from schools highlights perceived potential benefits, meeting this demand with limited resources can be challenging, and where the demand cannot be met, schools can potentially feel excluded or left-out. Finite funding resources can influence engagement practitioners’ decision to either opt for a greater depth of face-to-face, and arguably higher quality engagement with fewer schools, or to widen the outreach to a larger number of schools with shallower engagement ^85^. At KWTRP, our PAR approach has resulted in a compromise between the two positions, combining a greater depth of engagement with 5–6 schools per year, with less-intensive engagement with up to 37 schools. Our experience is that though schools often feel disappointed to shift from the annual rota of the 5–6 face-to-face schools, the disappointment is lessened through an opportunity for continued participation in less-intensive school engagement. The approach also enables the SEP team members to maintain contact with all schools as the rotation proceeds.

Hyder, Krubiner ^86^ argue that the longer a research institution works in a community, the greater the obligation for researchers to ensure greater benefits for host communities. However, they limit their discussion to benefitting communities through improving health infrastructure and boosting local economies through their presence in the community. The evolution of, and the demand for the SEP in Kilifi has shown that health research Institutions such as the KWTRP can draw from their existing human and science resources to benefit local schools through enhancing students’ science education experiences. As more KWTRP studies depend on schools for studying health and diseases, for example Abubakar, Kariuki ^87^ and Brooker, Okello ^88^, there may be a case for increasing benefits to local schools as a means of addressing of long-term community benefits. The experience of establishing a SEP suggests that engagement programmes such as the SEP can become spaces where community members are empowered to negotiate the terms and benefits of engagement and create mutually-beneficial initiatives.

## Conclusion

School engagement offers opportunities to draw from existing health research resources to benefit both researchers and schools. The benefits of engagement perceived by schools can create a demand for further and wider engagement which can ultimately contribute to the sustainability of SEPs. In our experience, including researchers, teachers and students in the design and implementation of school engagement through a PAR approach, can ensure that activities are responsive to participant views and locally appropriate.

## Disclaimer

The views expressed in this article are those of the author(s). Publication in Wellcome Open Research does not imply endorsement by Wellcome.

## Data availability

No data is associated with this article.
