# Supplementary material for: Evolution of a programme to engage school students with health research and science in Kenya
Source: Wellcome Open Res. 2019 Feb 28;4:39. [Version 1] doi: 10.12688/wellcomeopenres.15106.1 (PMC6419976; doi:10.12688/wellcomeopenres.15106.1)
Supplement: Peer review history

# Peer review: Reviewer response for version 1

**DOI:** 10.21956/wellcomeopenres.16482.r34973

**Published:** 2019-04-11

**Authors:** Kathryn Woods-Townsend

**Affiliations:** 1 Faculty of Social, Human and Mathematical Sciences, Southampton Education School, University of Southampton, Southampton, UK

This is an insightful and extremely relevant article, discussing the evolution of a programme to engage school students with health research and science in Kenya.

 The authors set the context for the development of this programme – the impetus to develop it, the local, national and international contexts in which their and similar school engagement programmes are situated, the development process and timeline and the lessons learnt for dissemination to others.

 Understanding the need for such engagement programmes in schools in differing localities, with all the inherent challenges, barriers and opportunities that each environment brings is fascinating. I have no doubt that there are many shared experiences to learn from but also unique insights brought to bear when considering different communities.

 The commitment of KWRTP to developing such a well-considered approach is to be applauded, particularly the vision from the start to understand the best ways to develop such a programme that would consider the needs of differing stakeholders. Taking a strong methodological approach and considering evaluation from the outset is also a strength. Appreciating their role in the community as a well-funded state of the art research facility and committing to providing opportunities to support the communities for whom research is being conducted, but also in supporting the training of the next generation of scientists is commendable.

 This article included a well-written review of various school engagement approaches currently being undertaken across the world, with a consideration of how differing motivations and local contexts shape the evolution of these approaches.

 I was interested in the motivation to develop a SEP in order to meet local demand for support in schools to advise school students on careers and also to meet the desire of KWRTP in supporting raising school students’ aspirations towards science and research-related careers and also in the wider aim of seeing school students as agents of change in their families and communities. There was a well thought through cycle of development, delivery, evaluation and refinement over several years and the PAR approach has obviously worked well and ensured buy-in from a variety of stakeholders. This ensures that the SEP which is offered to schools is more likely to meet their needs and be practically possible. This also offers a way to address challenges and come up with solutions together, so that all stakeholders are a part of that process and feel ownership of the ‘final’ programme.

 I have a few queries/comments – which maybe being addressed in subsequent articles, but I would be interested in understanding in the context of this article also.

 I would be interested in knowing more about the detail of the activities delivered to school students. I’m interested in where the engagement of parents is, in the first paragraph talking about the PAR approach, parents are mentioned, and it is stated that the activities were well received by parents, but I’m not clear if parents’ views were sought in the development of the activities, or during the development and delivery of subsequent cycles. If school students are to be seen as agents of change in their families, then some way of ensuring input from families is critical.

 Although the SEP was developed to address engage school students with health research, I didn’t get the sense from the article that the health aspect of the research had been capitalised on particularly. School students are very motivated to understand about themselves and their health, and so using the research as a hook to engage the students directly – maybe even with a view to changing their behaviours - is very powerful and makes this research so much more tangible to them than research which is more abstract for them. It maybe that this is just inherent in your approach, but it didn’t come across in this article.

 What is your approach for a longitudinal follow-up of the students who were involved in this project? E.g. the students who took part in the first cycle – have a higher proportion of these gone on to further study/engagement with science/research related jobs/careers?

 Finally, this is obviously a huge success in your area and offer such a rich engagement opportunity for the schools and students, do you envisage it being challenging to maintain the enthusiasm from the schools - if only 5/6 schools can take part in face to face engagement with you, that is effectively a 7 year cycle of the more intensive engagement? Does the online engagement sufficiently maintain the buy-in from schools?

 I am very interested in reading more about the evaluation and impact of both the face-to-face engagement and the online engagement.

I have read this submission. I believe that I have an appropriate level of expertise to confirm that it is of an acceptable scientific standard.

# Peer review: Reviewer response for version 1

**DOI:** 10.21956/wellcomeopenres.16482.r34974

**Published:** 2019-03-14

**Authors:** Bernard Appiah

**Affiliations:** 1 Research Program on Public and International Engagement for Health, Department of Environmental and Occupational Health, School of Public Health, Texas A&M University, College Station, TX, USA; 2 Centre for Science and Health Communication, Accra, Ghana

The authors of the manuscript have done a great job of providing an overview of community engagement/public engagement with a specific focus on schools engagement. Overall, the authors provide useful information on the SEP being implemented by the KEMRI-Wellcome Trust Research Programme (KWTRP) in Kilifi, Kenya, its history, development and scale-up.

 Key strengths of the manuscript include the following:

1. Description of international experience of engagement with adequate references, which helps put the KWTRP in context.
2. Use of participatory action research to document key cycles of the KWTRP SEP experience, which addresses gaps in public engagement research such as those that lack theoretical frameworks.

I highly recommend that this article is accepted for indexing following minor clarifications/changes below.

**First,** given that students have different age categories, it may be helpful for the authors to indicate roughly the age category that the KWTRP SEP focuses on. For example, some countries have two main types of secondary schools: junior secondary school and senior secondary school. Thus, including the age category of the students may help readers to know the type of secondary school KWTRP SEP targets.  

**Second**, under “The general aims and approaches to community engagement in LMICs”, the authors provide important goals of community engagement. While the goals are great, their presentation seems inconsistent with parallelism. For example, whereas “These goals comprise … broadly protecting communities in research… minimising possible exploitation” looks good, “These goals comprise… increase the likelihood that research will generate fair benefits locally…. minimise community disruption” seems inconsistent. Thus, the authors should consider making the presentation consistent.

**Third**, the authors describe a 10-year SEP experience (“the development of the Kilifi SEP involved three cycles of PAR over a ten-year period”) but using the three cycles (PAR cycle 1, PAR cycle 2 and PAR cycle 3), it becomes clear that it started in 2009 and ended in 2017, which makes it 9 years. This may need a little clarification.

**Fourth**, under PAR cycle 2:2010-2012, it is indicated that “an estimated 1000 students visiting the KWTRP for engagement activities, with more students interacting with researchers through researcher visits to schools to give career and inspirational talks”. Perhaps, if feasible, a rough estimate of number of researcher visits to schools could also be given. This way, researchers (readers) may be more informed about the “quantity” of the engagement.

**Fifth**, under “International experience of school engagement”, the authors include a sub-title “researcher gains from school engagement” but a related sub-title on how schools could also gain is not presented although there are paragraphs that provide that information. The authors should consider including a sub-title on how schools could benefit or consider deleting the sub-title “researcher gains from school engagement”. If the “researcher gains from school engagement” is deleted, the authors should consider modifying the sub-title directly under the section as “Engagement between researchers and schools: benefits and approaches” instead of “Engagement between researchers and schools”. In addition, in the last paragraph of this section, Kenya is described as “a low-income country”. I think this may need to be lower middle-income country, as per the link.

**Sixth**, it appears that although the authors indicate in the abstract that “We conclude with a discussion about the challenges, benefits and lessons learnt from the SEP implementation and scale-up in Kilifi, which can inform the establishment of SEPs in other settings”, it seems to me that the challenges were not adequately discussed. For example, other than meeting the demand with limited resources, what potential challenges did the authors face in implementing the project? Or what challenges did the researchers and the schools face? And how did they navigate them? I think making the answers to these clear in the discussion may complement the benefits the discussion seems to slightly focus on.

I have read this submission. I believe that I have an appropriate level of expertise to confirm that it is of an acceptable scientific standard.

# Peer review: Reviewer response for version 1

**DOI:** 10.21956/wellcomeopenres.16482.r34972

**Published:** 2019-03-13

**Authors:** Nicola Desmond

**Affiliations:** 1 Liverpool School of Tropical Medicine, Liverpool, UK; 2 Malawi-Liverpool-Wellcome Trust Clinical Research Programme, Blantyre, Malawi

This article on the staged development of a combined face-to-face and less intensive schools engagement programme established by KEMRI in Kenya over the last six years was a fascinating read. The authors provide clearly referenced detail on the rationale for the establishment of such a programme as well as a replicable step-by-step guide to the staged approach, drawing on tools of Participatory Action Research to ensure collective engagement and evaluation as the programme progressed. I really enjoyed reading this and herald it as an example of good practice in science communication more generally and of schools engagement in particular.  

 The authors rightly highlight the fact that it is rare for such Science Communication programmes in low resource settings to publish actual practice but this is something all such institutions should aim for as it adds real value to understanding what works and what doesn't within public/community engagement in low resource settings as well as the ethical and moral responsibilities international research institutions located in such settings have to the communities they work alongside. Of course, making decisions as to what should be the approach and where to target engagement for programme-wide science communication approaches is often challenging, given the number of different priorities expressed by different publics. In this paper, the authors highlight the importance of engagement from scientific researchers within these institutions to the success of the programme, and I agree this is fundamental. Perhaps this was not the case in the Kenyan setting, but the challenges inherent in engaging busy researchers in public/community engagement activities cannot be ignored. I would have liked to have heard more about how their buy-in was achieved in this instance and how representative this was across disciplines and areas of research. This may be something to consider more generally in considering the role and utility of science communication and engagement within these institutions.  

 I really enjoyed this paper overall and look forward to more publications evaluating the impact of this school engagement programme.

I have read this submission. I believe that I have an appropriate level of expertise to confirm that it is of an acceptable scientific standard.
